# Shape Optimization of Bone-Bonding Subperiosteal Devices with Finite Element Analysis

**DOI:** 10.1155/2017/3609062

**Published:** 2017-12-17

**Authors:** Takeshi Ogasawara, Masayoshi Uezono, Kazuo Takakuda, Masanori Kikuchi, Shoichi Suzuki, Keiji Moriyama

**Affiliations:** ^1^Department of Maxillofacial Orthognathics, Graduate School of Tokyo Medical and Dental University, 1-5-45 Yushima, Bunkyo-ku, Tokyo 113-8510, Japan; ^2^Japan Society for the Promotion of Science, 5-3-1 Kojimachi, Chiyoda-ku, Tokyo 102-0083, Japan; ^3^Institute of Biomaterials and Bioengineering, Tokyo Medical and Dental University, 2-3-10 Surugadai, Kanda, Chiyoda-ku, Tokyo 101-0062, Japan; ^4^International Center for Materials Nanoarchitectonics, National Institute for Material Science, 1-1 Namiki, Tsukuba, Ibaraki 305-0044, Japan

## Abstract

Subperiosteal bone-bonding devices have been proposed for less invasive treatments in orthodontics. The device is osseointegrated onto a bone surface without fixation screws and is expected to rapidly attain a bone-bonding strength that successfully meets clinical performance. Hence, the device's optimum shape for rapid and strong bone bonding was examined in this study by finite element analyses. First, a stress analysis was performed for a circular rod device with an orthodontic force parallel to the bone surface, and the estimate of the bone-bonding strength based on the bone fracture criterion was verified with the results of an animal experiment. In total, four cross-sectional rod geometries were investigated: circular (Cr), elliptical (El), semicircular (Sc), and rectangular (Rc). By changing the height of the newly formed bone to mimic the progression of new bone formation, the estimation of the bone-bonding strength was repeated for each geometry. The rod with the Rc cross section exhibited the best performance, followed by those with the Sc, El, and Cr cross sections, from the aspects of the rapid acquisition of strength and the strength itself. Thus, the rectangular cross section is the best for rod-like subperiosteal devices for rapid bone bonding.

## 1. Introduction

In the last decade, bone-bonding devices have become commonly used as dental implants [1], bone fixation plates [2], artificial temporomandibular joints [3], and microscrews for orthodontic treatment [4] in the fields of oral and craniomaxillofacial surgery. However, since these devices require drilling into the bone tissues for fixation and since critical tissues such as nerves, blood vessels, and tooth germs are embedded in the bone tissues of the craniomaxillofacial region, their application inevitably involves serious risks of injury to these tissues. [5, 6]. Among the several methods for reducing these risks, a less invasive bone-bonding device called Onplant (Nobel Biocare, Gothenburg, Sweden) was developed [7].

Onplant, a bone-bonding device for orthodontic treatment, is not implanted into bone tissue with screws; it is placed onto the bone surface and is osseointegrated onto the underlying bone after a healing period [8]. Apparently, it does not incur the abovementioned risks; however, it requires a long healing time of 3-4 months for osseointegration. To shorten the healing time, a hydroxyapatite collagen bone-like nanocomposite (HAp/Col) [9] coating was developed [10]. HAp/Col-coated Ti rods have achieved rapid osseointegration onto rat calvarium in as early as 4 weeks. The measured bone-bonding strength is 16.4 N with an osseointegrated Ti rod with a length of 6.0 mm and a circular cross section.

For the realization of this prototype in clinical use, we have to improve the device to attain a greater bone-bonding strength within 4 weeks after surgery. Noting that a clinical bone-bonding device for orthodontic treatment has a strength of approximately 200 N [11], a length of 80 mm is required for a circular-rod-like device, but it is impossible to apply such long device to the maxilla and mandible, which have complex bone-surface morphologies [12]. In contrast, in our previous pilot study, HAp/Col-coated semicircular Ti rods with the same length were demonstrated to have a greater bone-bonding strength than that of circular Ti rods [13]. Thus, an improvement in the cross-sectional geometries of the rods might increase the bone-bonding strength; however, the injudicious repetition of animal experiments for examining various geometries cannot be ethically justified.

Here, we adopt a finite element (FE) analysis to overcome this difficulty. An FE analysis is a numerical method that calculates the stresses and determines the mechanical behavior of complex structures [14]. Design optimization utilizing this method has already been attempted, and its effectiveness was demonstrated [15]. Thus, the objective of this study is to evaluate the bone-bonding strength of Ti rods with various cross-sectional geometries by using an FE analysis and to determine the most promising candidate for future investigations.

## 2. Materials and Methods

### 2.1. Model Creation

**Figure 1:**
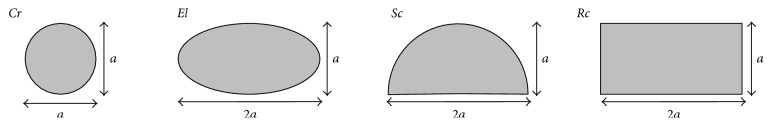
Assumed cross sections for the rod-like subperiosteal device.

The practical shapes of subperiosteal bone-bonding devices are severely restricted owing to demanding clinical requirements. Above all, their heights should be very small such that they can be placed onto the bone surface without serious damage to the periosteum. Further, as it is necessary that rapid osseointegration is established as early as within 4 weeks to match the needs of orthodontic practice [10], a smaller width is preferable because it enables osteogenic cells from the surrounding periosteum to reach around the device and build osseous tissues there, thereby enabling rapid osseointegration. Therefore, the devices were assumed to have a rod-like shape in this study. In this case, the cross-sectional geometries of the devices have significant effects on the bone-bonding strength. Therefore, we selected distinctive cross sections for the rods: circular (Cr), elliptical (El), semicircular (Sc), and rectangular (Rc), as shown in Figure 1; the heights of all sections were assumed to be the same and are denoted by* a*, and the widths of the wider sections other than a circular section are denoted by 2*a*. Moreover, the geometry of the underlying bone was assumed to have a plate shape for simplification.

**Figure 2:**
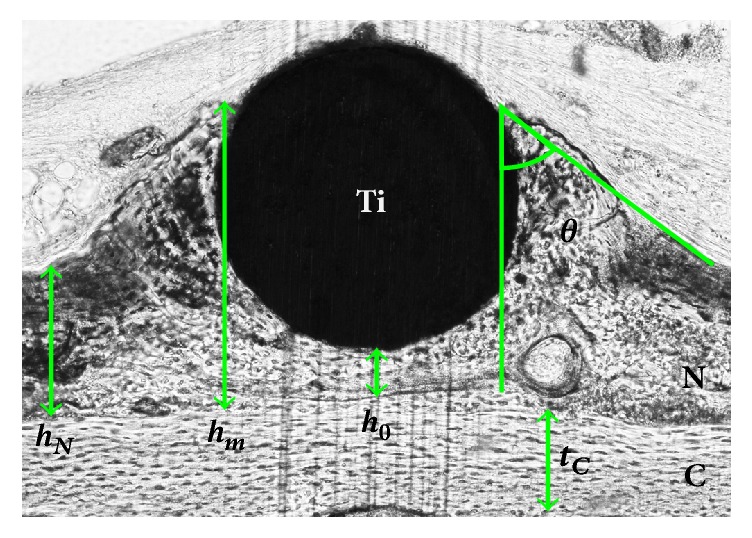
Characteristic values from a histological image: *t*_*C*_: thickness of the calvarium (C), *h*_*N*_: height of the newly formed bone (N), *h*_*m*_: maximum height of the newly formed bone, *h*_0_: height of the newly formed bone under the Ti rod (Ti), and *θ*: contact angle of the newly formed bone.

During the healing process following placement of the device, osteogenic cells migrate from the surrounding periosteum in the vicinity of the device and form new bone tissue. At 4 weeks after operation, the new bone tissue covers the entire bottom surface and the lower halves of the lateral surfaces of rods with a circular cross section [10]. Similar bone formation was also observed for rods with a semicircular cross section [13]. Hence, we assumed that this new bone formation occurs for all rods irrespective of their cross sections. Moreover, the bonding geometry between the device and the bone as well as the underlying bone thickness was created on the basis of a morphometric analysis of histological images of a rod with a circular cross section using image analysis software (Image J: Toronto Western Research Institute, Toronto, Canada).  Figure 2 and Table 1 present the five characteristic values obtained—*t*_*C*_: the thickness of the calvarium (C), *h*_*N*_: the height of the newly formed bone (N), *h*_*m*_: the maximum height of the newly formed bone, *h*_0_: the height of the newly formed bone under the Ti rod (Ti), and *θ*: the contact angle of the newly formed bone. For cross sections other than the circular one, we generated the bonding geometries from these characteristic values.

The amounts of the newly formed bone tissue around the device increase with time. In order to simulate such a phenomenon, groups of FE models with various heights of newly formed bone were constructed for each cross-sectional shape. In these groups, *h*_*m*_ was varied from 345 *μ*m to 525 *μ*m in 20 *μ*m steps while the other characteristics remained constant.

### 2.2. Finite Element Models

**Figure 3:**
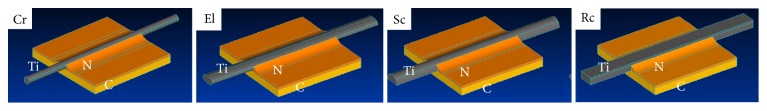
Finite element models of the four groups. The models consist of the calvarium (C), newly formed bone (N), and a Ti rod (Ti). The Ti rod has four different cross-sectional geometries: Sc: circular, El: elliptical, Sc: semicircular, and Rc: rectangular (*h*_*m*_ = 445 *μ*m).

Three-dimensional FE models simulating the mechanical test performed in a previous study [10] were created with the use of integrated FE analysis software (Femap with NX Nastran: Siemens PLM, USA & Canada) using a personal computer (ThinkCentre M92p, Lenovo, Hong Kong), as shown in Figure 3. For the cross sections of the rods, we set *a* = 0.5 mm, the length of the rod equal to 12.0 mm, and each length of a protruding part of a rod equal to 3.0 mm; these settings provide an identical correspondence between the model of a rod having a circular cross section and *h*_*m*_ = 445 *μ*m and the experimental samples. The models consisted of tetrahedrally shaped solid elements. The numbers of elements and nodes in each model were 198,160 elements and 219,545 nodes for the Cr group, 224,360 elements and 248,720 nodes for the Sc group, 214,240 elements and 237,276 nodes for the El group, and 228,400 elements and 253,583 nodes for the Rc group. These numbers were varied depending on *h*_*m*_. For example, the numbers with the maximum *h*_*m*_ of 525 *μ*m were as follows: 226,385 elements and 205,537 nodes for the Cr group, 245,196 elements and 223,194 nodes for the El group, 251,112 elements and 227,022 nodes for the Sc group, and 254,783 elements and 229,510 nodes for the Rc group. Moreover, the numbers with the minimum *h*_*m*_ of 345 *μ*m were as follows: 222,305 elements and 200,675 nodes for the Cr group, 230,076 elements and 206,742 nodes for the El group, 243,440 elements and 218,315 nodes for the Sc group, and 247,343 elements and 221,866 nodes for the Rc group.

All materials were configured to be homogeneous, isotropic, and linearly elastic. The mechanical properties utilized in the models are listed in Table 2. The rods were assumed to be made with the grade 2 Ti specified in ASTM F67 [16, 17]. The underlying bone was assumed to be the calvarium [18, 19], and the same properties were adopted for the newly formed bone. The yield strength from previous studies of conventional bone-bonding devices was employed [20]. The boundary conditions were determined to simulate the mechanical tests. The interface between the calvarium and the newly formed bone and that between the newly formed bone and the rod were assumed to be fully bonded. The bottom of the calvarium was fully constrained. The orthodontic force was evenly applied to both protruding parts of the rod, parallel to the calvarium surface and perpendicular to the long axis of the rod.

### 2.3. Estimation of the Bone-Bonding Strength

Making use of the above FE models, the bone-bonding strengths of the rods were estimated in the following steps.

(i) In the load-free state, the rod and bone tissue were assumed to be completely bonded since the rod is osseointegrated onto the bone. Further, the strength for the interface between the rod and the bone was assumed to be the same as the yield strength of the bone tissue itself.

(ii) The orthodontic force that causes fracture in the rod-bone specimen was estimated. Because of the linear nature of the problem, the magnitude of the fracturing force was calculated after dividing the yield strength by the maximum von Mises stress induced in the specimen under the application of a unit load. In the actual analyses, the maximum stresses were identified at the rod-bone interface. Thus, the load causing fracture at the rod-bone interface was estimated in this step.

(iii) After fracture at the interface, the bonding conditions were changed to stress-free conditions for the tensile part of the interface and friction contact conditions for the compressive part. The friction constant was initially assumed to be 1.0; however, it was subsequently observed that this value had little effect on the stress distributions.

(iv) The orthodontic force causing further fracture of the rod-bone specimen was estimated. As in step (ii), the magnitude of the fracturing force was calculated. In the actual analyses, a load that is smaller than that obtained in step (ii) induced fracture in the bone covering the rod, which means that bone fracture immediately followed fracture at the interface; then, the rod debonded from the bone.

## 3. Results

**Figure 4:**
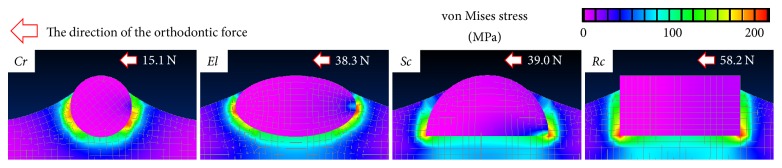
von Mises stress contours at the load causing the initial interface fracture (*h*_*m*_ = 445 *μ*m).

First, we estimated the von Mises stresses at the load causing the initial interface fracture (Figure 4). For all rods, the stresses induced in the calvarium and rods were relatively small. The maximum stresses were observed in the newly formed bones. Hence, for all rods, the initial fracture occurred at the bottom interface between the rod and the bone. The contours are symmetric, and the maximum stresses are identified at two locations. However, the interfacial stresses are tensile on the right side and compressive on the left side. Consequently, the interfaces become open and stress-free on the right side and become a friction contact on the left side.

**Figure 5:**
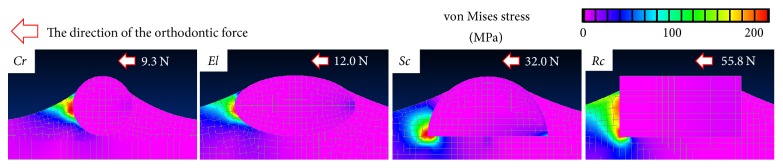
von Mises stress contours at the load causing bone fracture following the initial interface fracture (*h*_*m*_ = 445 *μ*m).

Second, we estimated the von Mises stress at the load causing bone fracture following the initial interface fracture (Figure 5). For all rods, bone fracture occurred on the left side of the newly formed bones, which were partly covering the lateral surfaces of the rods. Further, since these bone fracture loads were smaller than those corresponding to the interface fracture loads, the rods debonded from the bone surfaces immediately after interface fracture, and these interface strengths are the bone-bonding strengths.

**Figure 6:**
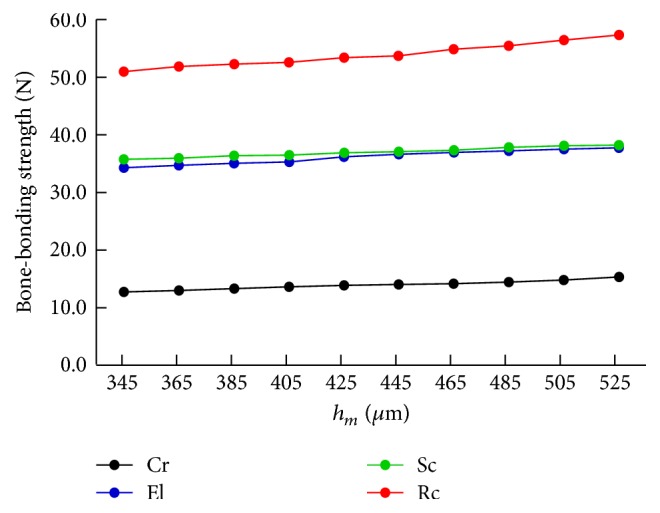
Changes in the bone-bonding strengths during the progression of bone formation.

The changes in the bone-bonding strengths during the progression of bone formation are shown in Figure 6. Since the rates of bone formation differ for rods with different cross sections, we cannot compare the strengths of these rods for the same *h*_*m*_. Hence, we examined the changes in the bone-bonding strength according to the changes in *h*_*m*_, which increases as bone formation progresses. Figure 6 indicates that the estimated bone-bonding strengths increase as *h*_*m*_ increases, and the rod with the rectangular cross section always exhibits the largest strength, followed by the semicircular and elliptical cross sections and finally the circular cross section.

In all analyses, no error messages were reported in the output files.

## 4. Discussion

In our animal experiments [10], mechanical tests were carried out, in which a load was evenly applied to both protruding parts of a rod with a circular cross section, parallel to the calvarium surface and perpendicular to the long axis of the rod to mimic the orthodontic force. The mean bone-bonding strength was 16.4 N, and the mean value of *h*_*m*_ was 445 *μ*m. Micro-CT observations revealed that the rods were debonded from the bone with bone fracture on the compressive side of the newly formed bone partially covering the lateral surface of the rod. The FE model corresponding to this experiment was that of the Cr group with *h*_*m*_ = 445 *μ*m, and our analysis estimated the bond strength to be 15.1 N. It also predicted the location of the final bone fracture in the newly formed bone. Further, in our previous pilot animal experiments with a rod having a semicircular cross section [13], the mean bone-bonding strength was measured as 46.8 N with almost the same value of *h*_*m*_, and final bone fracture was observed on the compressive side of the newly formed bone. For this cross-sectional shape, our analysis estimated a strength of 39.0 N and final bone fracture on the compressive side of the newly formed bone. Thus, the estimation method in this study precisely predicted the final fracture mode and bone-bonding strength with an accuracy of 92.1% for a circular cross section and 83.3% for a semicircular cross section. Although the accuracy decreased for the semicircular cross section, the estimation method at least successfully showed a marked increase in the bone-bonding strength by changing the cross-sectional shape. Hence, the method for estimating the strength is considered to be validated.

The major cause of estimation errors might be attributed to the difficulties associated with the prediction of the fracture of the sample's structure. We adopted the yield strength criterion; however, plastic deformation continues after yielding, and debonding between the rod and the bone would actually occur under greater loading. In addition, two assumptions utilized in the FE analyses are also sources of estimation errors. First, the newly formed bone was assumed to have the same mechanical properties as those of the calvarium, but the mechanical properties of the newly formed bone produced in a cavity around dental implants have been reported to be different from cortical bone [21, 22]. However, the bone observed in these studies apparently has a woven bone-like structure, whereas the newly formed bone produced under the periosteum had a lamellar bone-like structure [10, 13]. Furthermore, many previous FE analysis studies did not consider the existing newly formed osseointegrated bone tissue in their models and thus implicitly assume that their mechanical properties are those of cortical bone [23–25], or they consider the newly formed bone tissue and assume that its mechanical properties are those of cortical bone [26, 27]. Therefore, we assumed that the newly formed bone and calvarium have the same mechanical properties. Second, the Ti rods and newly formed bone were assumed to be fully bonded. In the case of dental implants, the contact between the implant and the bone is lower than 50% in animal studies [28]. However, in the case of the HAp/Col-coated Ti rod, the rod surface is clearly divided into a fibrous-tissue contact region and a bone contact region, in which histological observations showed that the rods were fully bonded to the newly formed bone [10]. Therefore, we assumed complete bonding of the rods to the newly formed bone.

There exist inherent difficulties in the estimation of the bone-bonding strengths; however, even though the rates of bone formation differ for rods with different cross sections, the conclusion for the optimal shape would not be affected. As shown in Figure 6, the estimated bone-bonding strengths increase as *h*_*m*_ increases. The most significant feature of this result is the fact that the order of the strengths among the four cross sections is independent of the variation in *h*_*m*_. This means that a rod with a rectangular cross section is the most favorable since it has the greatest bone-bonding strength, and it attained the required bone bonding the fastest. The next favorable cross sections are the semicircular, elliptical, and circular cross sections, in that order.

The reason for why the rectangular cross section is the most favorable is as follows. For the rod-like shape of the device, elementary beam theory predicts its macroscopic deformation [29]; the deformation during the mechanical test is analogous to four-point bending, in which the inner two points correspond to both edges of the calvarium sample and the outer two points are the loading points in the test for measuring the strength. With this type of deformation, the amount of flexure in the rod is inversely proportional to the second moment of area of the rod *I*, which is a characteristic parameter of the cross section; the respective formulas and values are summarized in Table 3. Since the rectangular cross section has the maximum *I*, the flexure in the rod is the smallest among the four cross sections. On the basis of these qualitative analyses, elastic continuum mechanics predicts the pattern of the stress distribution in the newly formed bone around the rods; the maximum stress is generated at the edge of the bone sample. Since the rectangular rod has the smallest flexure at this place, the smallest stresses are induced; thus, a rod with this cross section has the greatest resistance to the load [27].

The potential limitations of our study are related to the status quo of computer simulation technology. A linear elastic analysis was assumed for simplicity; however, bone tissues are anisotropic and inhomogeneous [30]. Hence, nonlinear elastic-plastic analyses are desirable. Moreover, biological aspects should be considered in the optimization process. In particular, the upper sharp edges of the device inevitably contact the soft tissues [7], and they yield risks such as soft-tissue rupture and/or blood-flow obstruction. Adequate modifications of the cross-sectional rod geometries are necessary, and the device's design should be validated in animal experiments.

## 5. Conclusions

This is the first study related to the estimation of the bone-bonding strength of a subperiosteal device with an FE analysis. The rectangular cross section is the most favorable for a rod-like subperiosteal device.

## Figures and Tables

**Table 1:** Characteristic values of the model geometry.

	Value
*t*_*c*_ (*μ*m)	464.0
*h*_*N*_ (*μ*m)	165.0
*h*_*m*_ (*μ*m)	445.0
*h*_0_ (*μ*m)	63.0
*θ* (degrees)	64.0

**Table 2:** Material properties used in the finite element models.

Material	Young's modulus *E* (GPa)	Poisson's ratio *ν*	Yield strength (MPa)
Cortical bone	14.2	0.39	180
Newly formed bone	14.2	0.39	180
Titanium	105.0	0.37	275

**Table 3:** Formulas for the cross-sectional second moments of area of each cross section (*I)* and their values.

Groups	Cr	EI	Sc	Rc
*Formula*	$I=\frac{a^{4}\pi }{64}$	$I=\frac{a^{4}\pi }{8}$	$I=\frac{a^{4}\pi }{8}$	$I=\frac{2a^{4}}{3}$
*I*	0.003	0.025	0.025	0.041
